# Influence of Chitosan Purification on the Immunomodulator Potential of Chitosan Nanoparticles on Human Monocytes

**DOI:** 10.3390/polym16233390

**Published:** 2024-11-30

**Authors:** Bruno Alejandro Valades-Aguilar, Teodoro Iván Rivera-González, Raúl Rangel-López, Gabriel Luna-Barcenas, Moisés Ármides Franco-Molina, Cristina Rodriguez-Padilla, Diana Ginette Zárate-Triviño

**Affiliations:** 1 Laboratorio de Inmunología y Virología, Facultad de Ciencias Biológicas, Universidad Autónoma de Nuevo León, San Nicolás de los Garza 66455, NL, Mexico; bruno.valades.97@gmail.com (B.A.V.-A.); teorivera88@gmail.com (T.I.R.-G.); jrangelraul95@gmail.com (R.R.-L.); moyfranco@gmail.com (M.Á.F.-M.); crrodrig07@gmail.com (C.R.-P.); 2 School of Engineering and Sciences, The Institute of Advanced Materials for Sustainable Manufacturing, Tecnológico de Monterrey, Querétaro 76130, QRO, Mexico

**Keywords:** chitosan, chitosan purification, chitosan nanoparticles, macrophages, cytokines

## Abstract

The deproteinization of chitosan is a necessary purification process for materials with biomedical purposes; however, chitosan sourcing and purification methods can modify its molecular weight, deacetylation degree, and residual proteins. These factors affect the reactive groups that affect the immunomodulatory activities of cells, particularly macrophages and monocytes; considering this activity is key when developing successful and functional biomaterials. Here, two brands of chitosan were purified and used to synthesize nanoparticles to evaluate their immunomodulatory effect on monocyte and macrophage differentiation. Chitosan FT-IR showed bands related to its purification process, with increased OH group intensity. Nanoparticles (CtsNps) synthesized with purified chitosan were of a smaller size compared to those using unpurified chitosan due to the alkaline purification process’s shortening of the polymeric chain. At low concentrations (50 μg/mL), CtsNps showed a lower expression of CD80 and CD14, corroborating the differentiation effect of chitosan. Inducible nitric oxide synthase (iNOS) is related to a pro-inflammatory response and M1 macrophage polarization was detected in monocytes treated with purified and unpurified nanoparticles. Sigma-purified chitosan nanoparticles (CtsNps SigmaP), at 300 μg/mL, showed arginase production related to an anti-inflammatory response and M2 macrophage polarization. The chitosan purification process induces a shift in the polarization of macrophages to an anti-inflammatory M2 profile. This effect is concentration-dependent and should be further studied in each use case to favor the suitable biological response.

## 1. Introduction

Chitosan has been widely used as a drug delivery system in multiple tissues and in wound healing, and also in the delivery of vaccines and genes [1,2], and it is crucial to understand the inflammatory processes that it uses. In PBMCs, antigen-presenting cells such as monocytes and macrophages are the master regulators of inflammation; they are found in almost all tissues and are nearly the only cells capable of performing each step of inflammation, which is key to developing successful and functional nanobiomaterials [3].

Chitosan is one of the most widely used materials in biomedical science. This polymer is an inexpensive material extracted from insects, marine aquatic animals, and microorganisms such as fungi, yeast, and microalgae; it is produced by the partial deacetylation of chitin in an alkaline environment [4]. Structurally, chitosan is a linear polysaccharide composed of randomly distributed β-(1→4)-linked D-glucosamine on its deacetylated unit and N-acetyl-D-glucosamine on its acetylated unit. Depending on its source and the preparation procedures used, its molecular weight ranges from 300 to 1000 kD; the higher the molecular weight of chitosan, the less it will dissolve due to the formation of a large number of inter- and intra-molecular hydrogen bonds among its chains. With a deacetylation degree (DD) of 30–90%, the higher the DD, the greater its solubility in water due to the protonation of more amino groups in the chain. Chitosan is a polycationic polymer and the pKa value of its amino group is 6.5, meaning it is positively charged and soluble in acidic to neutral solutions [5,6,7].

The obtention of chitosan requires the removal of allergen proteins like tropomyosin by using an alkaline treatment as a purification method. These treatments modify the molecular weight and DD of chitosan, as well as its residual proteins [8]. These proteins generate an immune response in a living system; however, this response is limited and depends on the chitosan’s type, structure, and degree of purification, a process that is yet to be fully understood. Thus, to produce the desired effect, it is necessary to use high doses of chitosan nanoparticles (CtsNps), which in turn decreases the viability of peripheral blood mononuclear cells (PBMCs).

CtsNps are particularly useful due to their biocompatibility, biodegradability, hydrophilicity, relative nontoxicity, large surface-to-volume ratio, and physicochemical properties [9]. Size is also a major factor in their biological properties: nanoparticles smaller than 20 nm can permeate through blood vessels, the blood–brain barrier, and the epithelium of the stomach and can therefore be used as contrast agents to examine different organs during imaging. In contrast, larger nanoparticles (>200 nm) can be efficiently taken up by phagocytic cells due to their negative surface charge [10,11]. Chitosan nanoparticles are considered an inert biomaterial that can induce a mild reaction; however, they can induce a specific inflammatory response initiated by direct molecular recognition [12,13]. Therefore, chitosan needs to be purified in order to boost the immune response it generates at a low concentration and reduce the damage to PBMCs [14].

The physiological phagocytic activity of macrophages allows them to detect, recognize, and eventually capture any nanosystem cruising in their surroundings. Macrophages and dendritic cells share the same precursor and play key roles in immunity; however, modifying their behavior to achieve an optimal host response towards a device is still a challenge [15,16]. Macrophages have a polarization process dependent on foreign agents: M1 polarization is related to a pro-inflammatory environment and is associated with microbial and tumor activity, antigen presentation, and tissue injury; this profile is desirable within the immunological system and should be activated in antitumoral therapies. An anti-inflammatory scenario requires M2 macrophages, which are associated with immunoregulation and tissue remodeling; hence, materials with regeneration properties such as scaffolds, coatings, implants, and membranes should activate this polarization [17].

Chitosan activates macrophages to produce cytokines such as IL-1. Similarly, chitosan oligosaccharides induce the robust production of pro-inflammatory cytokines, such as TNF and IL-1β, by macrophages. However, one study did not find a significant increase in the production of these cytokines when dendritic cells were treated with chitosan. Given that chitosan is a diverse class of molecules manufactured from a variety of raw materials, different chitosans may induce different immune responses. Moreover, some researchers suspect that most chitosans are contaminated with varying levels of endotoxins, which would impact immune responses to them significantly [18].

The capacity of a biomaterial to enable a biological application is key when selecting the best option to use in any treatment. In this work, two brands of chitosan were purified and used to synthesize nanoparticles to evaluate their immunomodulatory effect on monocytes derived from human PBMCs in terms of their differentiation into M1 or M2 macrophages.

## 2. Materials and Methods

### 2.1. Chitosan Purification

Medium-molecular-weight chitosan from two different brands, Sigma-Aldrich (BCCK4007, St. Louis, MO, USA) with a DD of >75% and Bio Basic Canada (12352201, Markham, ON, Canada) with ≥90% DD, were dispersed at 1:1 (*w*/*v*) in NaOH 1 M for 2 h at 70 °C and filtered on a Buchner funnel, and washed with distilled water three times. Afterward, the chitosan pellet was recovered from the filter and dried at 40 °C for 12 h.

Once dehydrated, the sample was solubilized in acetic acid 0.1 M, filtered through paper, and pH was adjusted to 8.0. Precipitated material was recovered, washed with deionized water, and dried at 40 °C for 24 h [19].

### 2.2. Experimental Groups

To analyze changes in reactive groups induced by purification, four experimental groups of chitosan were used: Bio Basic Canada chitosan (QTS-CA), purified Canada chitosan (QTS-CAP), Sigma chitosan (QTS-Sigma) and purified Sigma chitosan (QTS-SigmaP).

To characterize and evaluate the immune response of chitosan nanoparticles, four experimental groups were used: Sigma chitosan nanoparticles (CtsNps-Sigma), purified Sigma chitosan nanoparticles (CtsNps-Sigma P), Bio Basic Canada chitosan nanoparticles (CtsNps-CA) and purified Bio Basic Canada chitosan nanoparticles (CtsNps-CAP).

### 2.3. Fourier-Transform Infrared Spectroscopy (FT-IR)

Chitosan samples were analyzed by Fourier Transform Infrared Spectroscopy (FT-IR). Samples were placed on an ATR crystal using a top plate and pressure arm. Data were analyzed with the software PERKIN ELMER spectrum (S20 Series Software) [20].

### 2.4. Chitosan Nanoparticles (CtsNps) Synthesis

Chitosan solution purified and unpurified at 1 mg/mL was made with acetic acid at 0.4 M by magnetic stirring for 1 h at 600 rpm. NaOH 1 M was used to achieve a 5.5 pH and the solution was centrifuged for 10 min at 800 rpm.

Synthesis was carried out by constantly stirring chitosan solution (1 mg/mL) at 600 rpm for 24 h by magnetic stirring and adding pentasodium tripolyphosphate (TPP) (2 mg/mL) by constant drip (1:5). Afterward, it was centrifuged at 1500 rpm for 15 min [21].

An opalescent solution was obtained and sterilized by ultraviolet light for 10 min [22].

### 2.5. Size and ζ Potential of Chitosan Nanoparticles

Nanoparticles were loaded in a capillary cell for spectrophotometry and analyzed at 25 °C, size was determined by dynamic light scattering. Laser doppler electrophoresis was used to determine ζ potential by NS90 nanosizer equipment (Malvern Instruments, Malvern, UK) [20].

### 2.6. Scanning Electron Microscopy

Chitosan nanoparticles were mounted uniformly on a carbon adhesive tape that was attached to a sample holder and analyzed by transmission electron microscopy at Centro de Investigación de Materiales Avanzados (CIMAV) [23].

### 2.7. Obtaining Peripheral Blood Mononuclear Cells (PBMCs)

Mononuclear cells were collected from human peripheral blood samples from 3 healthy individuals. Informed consent was obtained from all subjects involved in the study. Blood samples were taken by venous puncture and collected in tubes with heparin. A gradient of blood and polymorphoprep was made and centrifuged at 1600 rpm for 30 min at room temperature, according to manufacturer’s instructions (Polymorphprep, Serumwek Bernburg, Germany).

Peripheral blood mononuclear cells (PBMCs) were washed two times with 1× phosphate-buffered solution (PBS) for 10 min at 1600 rpm. Mononuclear cells were cultured in 6-well polystyrene plates at 37 °C on a 5% CO_2_ atmosphere for 24 h using Roswell Park Memorial Institute (RPMI) medium with 5% Fetal Bovine Serum (FBS) [24].

### 2.8. Confocal Microscopy

To visualize chitosan nanoparticles on the surface of the cell, Fluorescein Isothiocyanate (FITC) (0.1 mg/mL) was added, and to visualize chitosan nanoparticles inside of the cell, carboxyfluorescein succinimidyl ester (CFSE) at 10 mM was added, both before the addition of TPP. After 6 h of synthesis, PBMCs were treated with chitosan nanoparticles at 300 μg/mL for 12 h. DAPI was used to dye the nuclei of the cells for visualization.

The cells were visualized 12 h after treatment with a confocal microscope (Olympus BX61W1, BX61W1, Melville, NY, USA). Cells were acquired using 405 and 488 lasers to detect DAPI and FITC/CFSE using 20× objective and 10% laser potency. The images were performed in ImageJ software, (Zulu OpenJDK 13.0.6.) using the brightness and contrast tools to visualize DAPI, FITC, and CFSE signal [25].

### 2.9. Viability Assay In Vitro

After monocytes at 5 × 10^3^ cells/well were differentiated into macrophages, they were treated with chitosan nanoparticles at 50 or 300 μg/mL for 24 h. Then, cells were washed twice with PBS 1× and incubated with RPMI medium containing 0.15 mg/mL resazurin (100 μL/well). Resazurin reduction was determined by measuring the excitation at 530 nm and emission at 590 nm using a Varioskan Flash UV-VIS spectrophotometer (Waltham, MA, USA) plate reader. Each sample was analyzed in triplicate. Cells without treatment were used as a negative control and wells containing no cells were used as blank control [26].

### 2.10. Analysis of Expression of CD11c, CD14, CD80 and CD86 Markers

After monocytes were differentiated into macrophages, and the cells were treated with the nanoparticle experimental groups for 24 h. Afterward, the cell medium was removed using accutase from the plate to obtain the adhered cells; then, they were centrifuged for 5 min at 1200 rpm and resuspended in PBS 1×. Then, 2 μL of anti-human CD11c, CD14, CD80 and CD86 were added and incubated for 30 min at room temperature and covered from light. Subsequently, a wash was carried out with PBS 1× and the samples were analyzed by flow cytometry (BD accuriTMC6, Piscataway, NJ, USA) [27].

### 2.11. Determination of Phagocytic Activity of Macrophages

After monocytes were differentiated into macrophages, the cells were treated with the nanoparticle experimental groups for 48 h. Afterward, macrophages were centrifuged with PBS 1× at 1200 rpm/10 min and washed with RPMI-1640 without FBS at 1200 rpm/10 min.

To each of the macrophage samples, 1 mL of FITC-dextran solution, from Merck, was added and incubated for 6 h at 37 °C. Control samples were incubated with FITC-dextran at 4 °C and 37 °C. The samples were centrifuged at 1200 rpm/10 min and washed with PBS 1×. Samples were analyzed by flow cytometry using BD AccuriTMC6 equipment [28].

### 2.12. Analysis of Cytokine Secretion

The supernatant of treated cells was used to measure cytokine secretion by the BD Cytometric Bead Array Human Inflammatory Cytokines Kit for the determination of IL-1β, IL-6, IL-8, IL-10, IL-12p70, and TNF by flow cytometry using BD AccuriTMC6 equipment [29]. LPS was used as an inflammatory control [30].

### 2.13. Arginase and iNOS Detection by Immunofluorescence

Macrophages were treated with purified and unpurified CtsNps at 50 or 300 μg/mL for 72 h. Afterward, the medium was removed and cells were washed with 1× PBS for 5 min. Then, cells were fixed with 4% cold *P. falciparum* arginase (PFA) (300 μL per well) and incubated for 20 min under constant agitation. The PFA was removed and 1 mL of 2% FACS buffer was added; this process was repeated twice.

To perform the permeabilization of the cell, 300 μL of 0.1% triton buffer was added and the plates were incubated with constant stirring at room temperature for 20 min; after this, cells were washed with 2% FACS buffer at room temperature for 15 min (constant stirring). To visualize inducible nitric oxide synthase (iNOS) and arginase, nitric oxide synthase was blocked by adding 300 μL of 2% FACS buffer for 25 min at room temperature with continuous agitation [31].

Then arginase and iNOS primary antibody (Abcam, USA) were added to each well (1:500) and the culture plates were incubated for 2 h at room temperature and then washed thrice with 2% FACS under agitation for 15 min. After this, the secondary antibody (Abcam, Cambridge, UK) was added in a ratio of 1:100 (PE to mark arginase and Texas Red to mark iNOS), and the culture plates were incubated for 2 h, rewashed with 2% FACS and DAPI was added and incubated for 30 min; finally, it was washed with 2% FACS to remove excess labeling and the samples were analyzed using confocal microscopy (Olympus BX61W1) [32].

### 2.14. Statistical Analysis

All the statistical analyses were performed using the software included in Biorender.com. All the data are expressed as mean ± standard deviation. Statistical significance was determined using an analysis of variance (*p* < 0.05). All the experiments were performed in triplicate.

## 3. Results

### 3.1. FT-IR Analysis of Chitosan Purified and Unpurified

**Figure 1:**
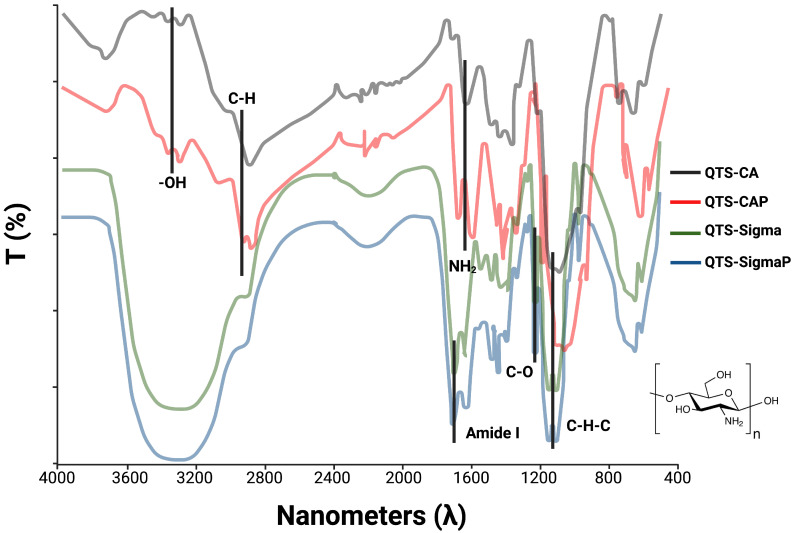
FT-IR absorption spectroscopy corresponding to purified and unpurified chitosan. QTS-CA and QTS-CAP showed splicing on a double group NH_2_. QTS-Sigma showed splicing on QTS-SigmaP on C-H groups. QTS-CAP and QTS-SigmaP showed splicing on OH groups. All samples showed splicing on amide I.

FT-IR spectrum of Bio Basic Canada (QTS-CA), purified Bio Basic Canada chitosan (QTS-CAP), Sigma chitosan (QTS-Sigma) and purified Sigma chitosan (QTS-SigmaP) showed characteristical bands of chitosan [33]. However, key differences were observed between QTS-CA and QTS-CAP with splicing between 1570 cm^−1^ to 1590 cm^−1^ related to a double group NH_2_; another splicing was observed at 1621 cm^−1^ to 1610 cm^−1^ associated with an amide I. On Sigma chitosan, C-H groups located at 3000 cm^−1^ and 2850 cm^−1^ showed splicing to 3090 cm^−1^ and 2900 cm^−1^ on the purified sample. The intensity of the band at 3400 cm^−1^ corresponding with OH groups increased for the purified samples (QTS-CAP and QTS-SigmaP) (Figure 1 and Table 1).

Water molecules can increase the widening of the bands at 3300 cm^−1^ by the purification process, Qts-CAP and Qts-SigmaP samples showed an increase in OH groups as well as a shift of NH_2_ and amide group to a lower wavelength due to hydrolysis alkaline process (Figure 1) [34]. According to Mina and her team, chitosan with a DD greater than or equal to 90% treated with an alkaline solution at 50% does not deacetylate significantly after 1 h, as seen on Qts-CAP [35]. Commercial chitosan from Sigma-Aldrich had a DD of greater than 75% and, based on FTIR measurements, there was no significant shift on sample Qts-SigmaP, suggesting a DD closer to 90% (Figure 1 and Table 1).

### 3.2. Characterization of Chitosan Nanoparticles (CtsNps)

Sigma chitosan nanoparticles (CtsNps-Sigma), purified Sigma chitosan nanoparticles (CtsNps-Sigma P), Bio Basic Canada chitosan nanoparticles (CtsNps-CA), and purified Bio Basic Canada chitosan nanoparticles (CtsNps-CAP) were measured by DLS. The samples had a size between 120 and 190 nm, a positive charge in ζ potential and a polydispersity index of 0.2–0.3 (Table 2).

**Figure 2:**
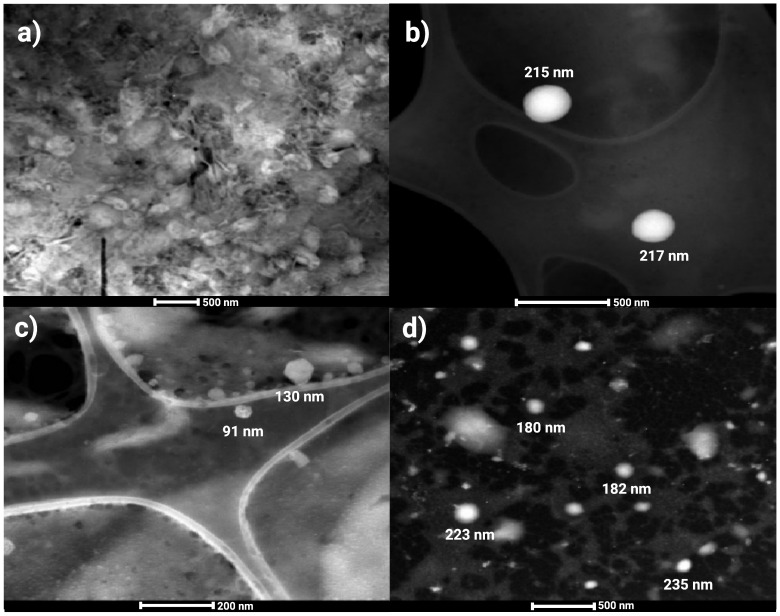
Transmission electron microscopy of chitosan nanoparticles. The spherical shape of the chitosan nanoparticles can be observed, as well as the size, which is less than 300 nm (scale of 500 nm). (**a**) Sigma chitosan nanoparticles, (**b**) purified Sigma chitosan nanoparticles, (**c**) Bio Basic Canada chitosan nanoparticles (scale of 200 nm) and (**d**) purified Bio Basic Canada chitosan nanoparticles.

Morphology was evaluated by transmission electron microscopy on CtsNps-CA, CtsNps-CAP, CtsNps-Sigma, and CtsNpsSigmaP. Figure 2a shows Sigma chitosan nanoparticles, Figure 2b shows purified Sigma chitosan nanoparticles, Figure 2c shows Bio Basic Canada chitosan nanoparticles (scale of 200 nm) and Figure 2d shows purified Bio Basic Canada chitosan nanoparticles. In all samples, the nanoparticles showed a spherical shape and a size minor of 300 nm.

It has been reported that the interaction charge can be effectively screened by electrolytes such as NaCl—the one used in the purification process. Charge screening weakens the repulsion charge between chitosan molecules, diminishing the size of the nanoparticles, which could explain the ζ potential changes in the purified samples [36].

The polydispersity index obtained in all samples was less than 0.3. Nanoparticles synthesized with purified chitosan decreased molecular weight and increased ζ potential, showing a smaller size in contrast to unpurified chitosan (Figure 2 and Table 2). Chitosan is deacetylated chitin, which is found in crustacean exoskeleton, fungal cell walls, and insects. Based on the source moisture, ashes, and amount of protein, lipid, yield, DD, and solubility change the physico-chemical behavior [37]. According to O’Callaghan and Kerry, this can be explained by the shortening of the polymeric chain due to the purification process [38,39]. It is also noted that molecular weight directly influences the size and ζ potential of nanoparticles which, in turn, affect their physical and biological properties [40].

### 3.3. Viability on Human Macrophages with Chitosan Nanoparticles

**Figure 3:**
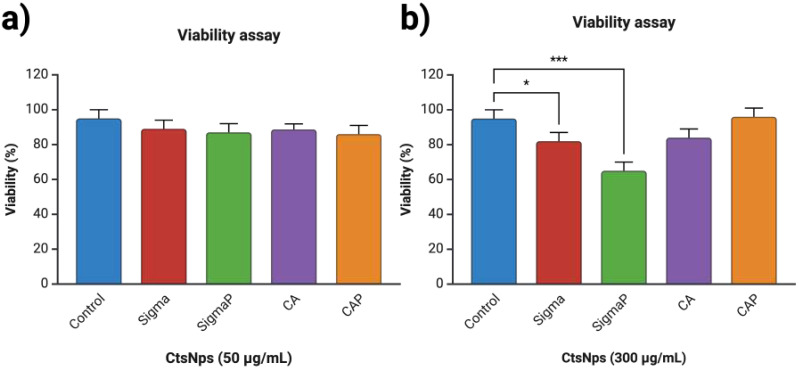
Cell viability on human macrophages with Sigma, SigmaP, CA and CAP nanoparticles at 24 h. (**a**) At 50 μg/mL there was no significant difference between the groups. (**b**) At 300 μg/mL there was a significant difference between Sigma and SigmaP compared to the control (* *p* < 0.05, *** *p* < 0.001).

Chitosan nanoparticles at 50 μg/mL on all samples do not significantly diminish the cell viability in comparison to the control (Figure 3a). At 300 μg/mL, Sigma and Sigma purified nanoparticles showed a decrease in viability, with SigmaP being highly significant in comparison to the control (Figure 3b).

Chitosan has been widely reported as non-toxic and suitable for drug delivery and has been designated as Generally Recognized As Safe (GRAS) by the Food and Drug Administration (FDA). On a nanometric scale, however, they have shown cytotoxic effects with concentrations between 0.125, 0.25, and 0.5 mg/mL on several reports [41]. Nanoparticle size does affect their cytotoxicity: sizes smaller than 200 nm have less cytotoxicity with viability greater than 90% [42]. As seen in Figure 3 and reported in Table 2, the concentrations were lower than 50 μg/mL and all types of nanoparticles had a sizer smaller than 200 nm, as there was no reduction in the viability on human PBMCs. However, the Sigma chitosan nanoparticles at 300 μg/mL showed a statistically significant reduction in viability and a greater reduction with the purified nanoparticles (Figure 3b), in comparison to the manufacturer Canada Bio Basic. This effect could be due to the different origins, chemical composition, and different sources and techniques of extraction, which influence the final properties of the chitosan [43], enabling them to be more suitable to phagocytosis.

### 3.4. Confocal Microscopy

**Figure 4:**
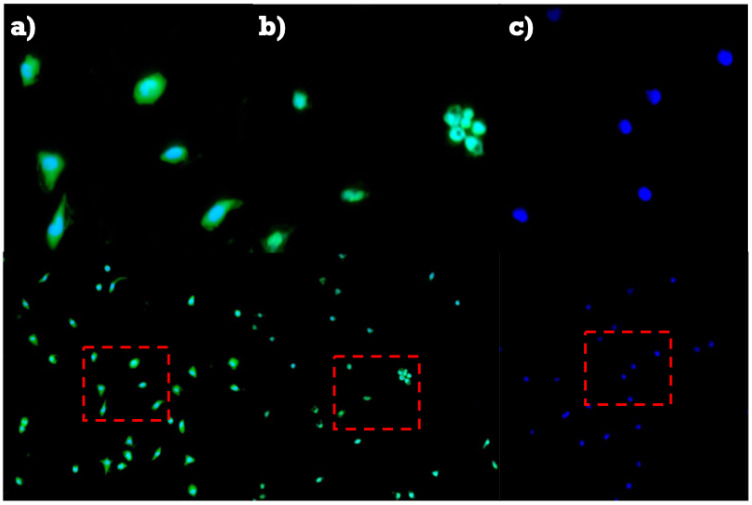
Chitosan nanoparticles conjugated with fluorophores interacting with human macrophages. (**a**) Green fluorescence of chitosan nanoparticles conjugated with FITC on macrophages surface, nuclei stained with DAPI as a localizer. (**b**) Fluorescence of metabolized CFSE conjugated with chitosan nanoparticles seen inside macrophages, nuclei signal was stained with DAPI. (**c**) Nuclei fluorescence with DAPI as control.

Chitosan nanoparticles conjugated with FITC are shown with green on the surface of macrophages (Figure 4a), whereas in chitosan nanoparticles conjugated with CFSE, the green fluorescence is seen inside of the macrophage (Figure 4b) showing that the nanoparticles have been metabolized. In all three images, nuclei are stained with blue fluorescent DAPI (Figure 4c).

A cell membrane is a multifaceted structure composed of lipids and proteins that provide an effective barrier against the majority of substances. Drug molecules must be able to attach or pass this barrier to achieve therapeutic activity. Chitosan nanoparticles encapsulating the fluorophore FITC can be seen attached to the cell membrane (Figure 4a). It is important to highlight that the degree of acetylation of chitosan influences its impact on cell adhesion and proliferation properties due to changes in hydrophobicity and the number of protonated groups available for cell adhesion. A greater degree of acetylation of chitosan used in films displayed increased hydrophobicity and a lower surface charge, leading to decreased adhesion [11].

The plasma membrane of mammalian cells has a net negative charge attributed to phospholipids having negative head groups. Hence, a cationic polysaccharide, such as chitosan can easily attach to the surface of cell membranes by electrostatic interactions, which results in enhanced cellular uptake. Previous studies have reported that molecular weight plays a critical role in the affinity of chitosan protein interactions; therefore, these parameters are essential for the immune response [44]. Chitosan nanoparticles encapsulating the green fluorophore CFSE can be seen inside the cell (Figure 4b). CFSE is an effective and popular means to monitor lymphocytes due to their covalent long-lived dye that fluoresces inside the cell [45], resulting in the nanoparticle being able to attach to the surface and enter the cell.

### 3.5. Expression of Macrophages

**Figure 5:**
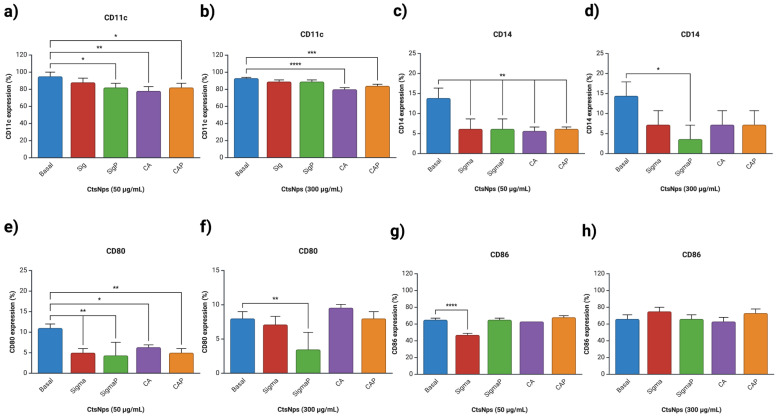
Evaluation of differentiation markers in macrophages treated with chitosan nanoparticles. Macrophages were treated with different CtsNps with a concentration of 50 μg and 300 μg for 72 h. (**a**,**b**) CD11c, (**c**,**d**) CD14, (**e**,**f**) CD80 and (**g**,**h**) CD86. Significant difference is observed in all markers except CD86 at 300 μg/mL (* *p* < 0.05, ** *p* < 0.01, *** *p* < 0.001 and **** *p* < 0.0001).

Macrophages were treated with 50 and 300 μg/mL of CtsNps (Sigma and Bio Basic Canada) purified and unpurified. A high expression of CD11c was observed on Sigma 50 μg/mL and Sigma and SigmaP at 300 μg/mL (Figure 5a,b), whereas a low expression of CD86 was only shown on Sigma at 50 μg/mL (Figure 5g).

Expression of CD14 and CD80 diminished significantly in all treatments at 50 μg/mL (Figure 5c,e), while at 300 μg/mL, only unpurified Sigma diminished their expression (Figure 5d,f).

Similar results were obtained with the use of a small molecule called iMAP2K3, which interacts with specific transcription factors, affecting the production of CD14 and CD80 [46,47].

Only macrophages treated with unpurified chitosan nanoparticles from Sigma at 50 μg/mL decreased the expression of CD86 compared with the control (Figure 5g), there was no difference with 300 μg/mL, and there was no difference in the expression of CD86 (Figure 5h).

Low expression of CD14 and high expression of CD80 and CD86 is related to M2 macrophage polarization, an anti-inflammatory seen on SigmaP at 300 μg/mL (Figure 5d–f,h) [48].

### 3.6. Determination of Phagocytic Activity

**Figure 6:**
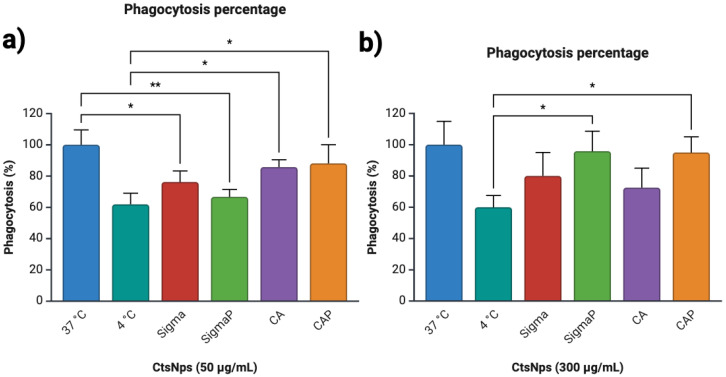
Phagocytic activity of macrophages treated with CtsNps. The macrophages were treated with two different concentrations of CtsNps, (**a**) 50 and (**b**) 300 μg/mL for 72 h, observing that the purified nanoparticles affect the phagocytic capacity of the cells (* *p* < 0.05, ** *p* < 0.01).

Macrophages were treated with 50 and 300 μg/mL of CtsNps (Sigma and Canada) purified and unpurified. All treatments decrease cells’ phagocytic activity in comparison to the control at 37 °C at 50 μg/mL, and SigmaP showed a minor phagocytic capacity at 67% (Figure 6a). purified Sig and CA CtsNps at 300 μg/mL showed a major phagocytic activity (92% and 82%) (Figure 6b).

M1 macrophage polarization is related to the inflammatory environment, phagocytic activity, and cytotoxicity. M2 macrophages are capable of lowering phagocytation [49], although some reports determined that exposing murine macrophages to different concentrations of CtsNps decreases their phagocytic capacity (Figure 6). This can be attributed to particle size: nanoparticles smaller than 275 nm are easily absorbed by the mucous membranes and the epithelial tissue and have a better distribution on intracellular traffic. The caveolae-mediated endocytosis pathway favors primarily particles with diameters smaller than 80 nm. Therefore, clathrin-mediated endocytosis and phagocytosis are potential mechanisms for the uptake of CtsNps [50,51].

Sigma-purified chitosan nanoparticles at 300 μg/mL had a greater decrease in viability (Figure 3b); this effect can relate to a greater percentage of phagocytosis; nevertheless, at the same concentration, Bio Basic Canada-purified nanoparticles did not diminish the viability, demonstrating that the cytotoxicity of nanoparticles depends on physico-chemical properties and the initial degree of DA before purification for each brand of chitosan.

### 3.7. Cytokine Analysis

**Figure 7:**
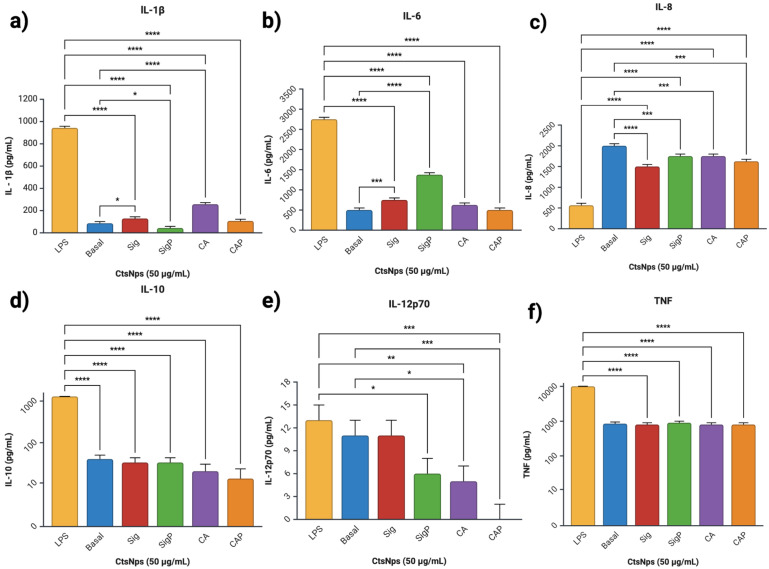
Profile of cytokines of macrophages treated with CtsNps. Macrophages were treated with concentrations of 50 μg/mL (**a**) IL-1β, (**b**) IL-6, (**c**) IL-8, (**d**) IL-10, (**e**) IL-12p70 and (**f**) TNF (* *p* < 0.05, ** *p* < 0.01, *** *p* < 0.001 and **** *p* < 0.0001).

**Figure 8:**
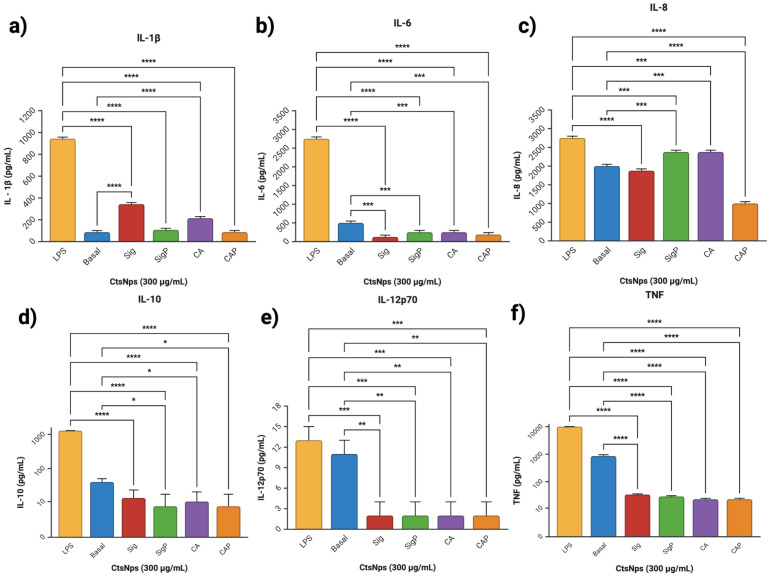
Profile of cytokines of macrophages treated with CtsNps. Macrophages were treated with concentrations of 300 μg/mL (**a**) IL-1β, (**b**) IL-6, (**c**) IL-8, (**d**) IL-10, (**e**) IL-12p70 and (**f**) TNF (* *p* < 0.05, ** *p* < 0.01, *** *p* < 0.001 and **** *p* < 0.0001).

Cytokine profiles obtained from macrophages treated with purified and unpurified chitosan nanoparticles showed an increased IL-1β on unpurified Sigma and CA at 50 and 300 μg/mL (Figure 7a and Figure 8a), IL-6 on Sigma, SigmaP and CA at 50 μg/mL (Figure 7b), compared to basal secretion, showing a major reactivity on cells treated with unpurified CtNps in contrast with cells treated with purified chitosan.

All CtsNps induce a primary immune response without reaching a pro-inflammatory profile due to low production of anti-inflammatory, mediating cytokines IL-10 in macrophages treated with purified CAP nanoparticles at 50 μg/mL (Figure 7d); this was also observable in IL-12p70 with SigmaP, CA and CAP nanoparticles at 50 μg/mL (Figure 7e).

CtsNps at 300 μg/mL induce a primary immune response without reaching a pro-inflammatory profile due to low production of anti-inflammatory, mediating cytokines IL-10 in macrophages treated on all samples (Figure 7d); this was also observable in IL-12p70 and all samples at 300 μg/mL (Figure 7e). These results suggest that purified nanoparticles can polarize an anti-inflammatory M2 phenotype. This is in agreement with Buchacher, who observed an M1 pro-inflammatory phenotype with high levels of IL-1β, IL-6, IL-12p70, and TNF, as well as regulation at high IL-10 as seen on Sigma 50 μg/mL (Figure 7a,b,d–f) [52].

### 3.8. Arginase and iNOS Detection

**Figure 9:**
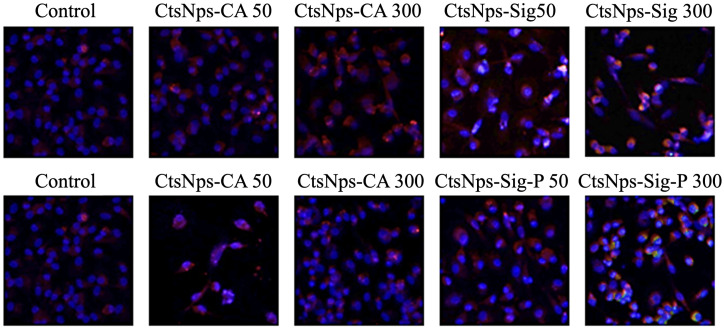
Arginase and iNOS detection in macrophages treated with CtsNps at concentrations of 50 and 300 μg/mL by confocal microscopy. Arginase production (green fluorescence) was only detected in macrophages treated with CtsNps SigmaP at 300 μg/mL. Higher production of iNOS (red fluorescence) was observed in macrophages treated with CtsNps; in comparison with the control group, the production of iNOS was not modified by the commercial and purified CtsNps.

Macrophages were treated with 50 and 300 μg/mL of CtsNps (Sigma and Bio Basic Canada) purified and unpurified. A low fluorescence of arginase (green) was detected in CtsNps SigmaP 300 μg/mL. iNOS production was increased with CtsNps (Purified and unpurified) in comparison with control; 300 μg/mL generated a higher production of iNOS (red fluorescence) (Figure 9).

Although there was no observed increase in pro-inflammatory cytokines and phagocytosis, the iNOS detection is related to pro-inflammatory M1 type. iNOS is a significant proinflammatory enzyme that results in the accumulation of nitric oxide and is a key factor in inflammation and tumor development [53]. However, inflammation is a constant state of balance: arginase production was seen in the SigmaP at 300 μg/mL group (Figure 9), suggesting an M2 polarization; this phenotypic cell mainly participates in the resolution of the inflammation process [54].

## 4. Conclusions

This study proves the importance of chitosan purification in the development of biomaterials. These results show that chitosan purification can diminish the size and modify the superficial charge of chitosan nanoparticles in contrast to unpurified chitosan nanoparticles, thus modulating the expression of molecules related to M1 or M2 macrophages. We suggest implementing the process of purification in any protocol involving chitosan of any kind.

## Figures and Tables

**Table 1:** Absorption bands in FTIR purified and unpurified chitosan.

Band (cm^−1^)	Group	Intensity
3300	OH	
3100–2850 *	C-H	Stretching
2800	C-H	
1650 *	Amide I	Bending
1550 *	NH_2_	
1200	C-O	Stretching
1010	C-H-C	

* Splicing.

**Table 2:** Average nanoparticle size, polydispersity and ζ potential of CtsNps-Sigma, CtsNps-SigmaP, CtsNps-CA and CtsNps-CAP.

Sample	Size	Polydispersity	ζ Potential
	nm	a.a	mV
CtsNps-Sigma	180	0.3	31.2
CtsNps-Sigma P	138	0.2	16.2
CtsNps-CA	177	0.3	3
CtsNps-CAP	126	0.3	7

Sigma chitosan nanoparticles (CtsNps-Sigma), purified Sigma chitosan nanoparticles (CtsNps-Sigma P), Bio Basic Canada chitosan nanoparticles (CtsNps-CA) and purified Bio Basic Canada chitosan nanoparticles (CtsNps-CAP).

## Data Availability

The original contributions presented in the study are included in the article, further inquiries can be directed to the corresponding author.
